# Significance of Soluble Lectin-Like Oxidized LDL Receptor-1 Levels in Systemic and Coronary Circulation in Acute Coronary Syndrome

**DOI:** 10.1155/2014/649185

**Published:** 2014-05-07

**Authors:** Tomofumi Misaka, Satoshi Suzuki, Nobuo Sakamoto, Takayoshi Yamaki, Koichi Sugimoto, Hiroyuki Kunii, Kazuhiko Nakazato, Shu-ichi Saitoh, Tatsuya Sawamura, Toshiyuki Ishibashi, Yasuchika Takeishi

**Affiliations:** ^1^Department of Cardiology and Hematology, Fukushima Medical University, 1 Hikarigaoka, Fukushima 960-1295, Japan; ^2^Department of Vascular Physiology, National Cardiovascular Center Research Institute, 5-7-1 Fujishiro-dai, Suita, Osaka 565-8565, Japan; ^3^Department of Cardiovascular Medicine, Ohara General Hospital, Ohmachi 6-11, Fukushima 960-8611, Japan

## Abstract

*Background.* Soluble lectin-like oxidized low-density lipoprotein receptor-1 (LOX-1) level is a novel biomarker for diagnosis of acute coronary syndrome (ACS); however, this level in the coronary circulation has yet to be examined. * Methods.* Twenty-seven consecutive patients with ACS and 40 patients with effort angina pectoris (EAP) undergoing percutaneous coronary intervention (PCI) had levels of soluble LOX-1 and LOX-1 index measured in paired blood samples from aorta (Ao) and coronary sinus (CS) just prior to the PCI. * Results.* We found positive correlations between soluble LOX-1 levels in the Ao and CS in both ACS and EAP patients (*P* < 0.01, for both). The soluble LOX-1 levels in the Ao and CS were higher in ACS than in EAP patients (*P* < 0.01, for both). The levels of soluble LOX-1 and LOX-1 index of the CS were significantly greater than those of the Ao in both ACS and EAP patients (*P* < 0.01, for both). Receiver operating characteristic curves for ACS detection demonstrated high sensitivity and specificity for the soluble LOX-1 and LOX-1 index with no differences between the Ao and CS. * Conclusions.* The present study showed that circulating soluble LOX-1 originates from coronary circulation and soluble LOX-1 and LOX-1 index are useful biomarkers for ACS.

## 1. Introduction

Biomarkers are a useful tool for the diagnosis and reflect the pathogenesis of coronary artery disease (CAD) [1]. Creatine kinase- (CK-) MB and troponins are derived from damaged cardiomyocytes, and their serum levels are elevated after the onset of acute myocardial infarction (AMI), making them practically useful for the diagnosis of AMI. Since high sensitive C-reactive protein (hs-CRP) is a representative marker of not only systemic but also vascular inflammation, serum hs-CRP levels are a predictive marker for cardiovascular diseases [2]. Moreover, patients with acute coronary syndrome (ACS) including ST segment elevated myocardial infarction (STEMI) and non-STEMI (NSTEMI) show higher levels of hs-CRP than those with stable angina pectoris [1, 3].

Although lectin-like oxidized low-density lipoprotein receptor-1 (LOX-1) was originally identified as a major receptor for oxidized low-density lipoprotein (LDL) in endothelial cells, LOX-1 has been shown to be expressed in various vascular cell components including smooth muscle cells and macrophages in response to vasoactive and atherogenic stimuli [4, 5]. In addition, a soluble form of LOX-1 has been detected in the systemic circulation, and its serum levels are elevated in ACS patients [6, 7]. Increased activities of serine proteases and matrix metalloproteinases cleave LOX-1 to promote the release of soluble LOX-1 into the systemic circulation from coronary atherosclerotic plaques [6]. However, there has been no report regarding the levels of LOX-1 in the coronary circulation.

Sato et al. have developed a system to measure the biological activity of the apolipoprotein B- (ApoB-) containing lipoprotein ligand based on binding to LOX-1, and they hypothesized that the activity of the LOX-1 ligand containing ApoB, termed LAB, might be more pronounced than oxidized LDL as a LOX-1 ligand [8]. Recently, a new assay system for soluble LOX-1 was established, and “LOX-1 index” was determined as [LAB × soluble LOX-1]. The LOX-1 index was even considered to possibly be a better marker than the LOX-1 ligand or soluble LOX-1 in some pathophysiological situations [9].

In the present study, we investigated the significance of soluble LOX-1, LAB, and LOX-1 index in the coronary circulation of patients with ACS and effort angina pectoris (EAP) by using paired blood samples from the aorta and coronary sinus.

## 2. Methods

### 2.1. Subjects

We included 27 patients with ACS who underwent emergent percutaneous coronary intervention (PCI) and 40 patients with EAP who received elective PCI from November 2008 to August 2009 in the present study. The diagnosis of NSTEMI was based on the Braunwald classification, and STEMI was diagnosed according to the refined ESC/ACC Committee criteria [10, 11]. The average time from the onset of ischemic chest pain to admission to hospital was 5.2 ± 3.5 hours (range 2 to 18 hours). Changes in ST-T segment and T-wave were present in all ACS patients. We excluded ACS patients with cardiopulmonary resuscitation before admission and patients with apparent inflammatory diseases, cerebrovascular diseases within 3 months, and other significant heart diseases. The investigation conformed with the principles outlined in the Declaration of Helsinki. Written informed consent was obtained from all patients, and the study protocol was approved by the Ethical Committee of Fukushima Medical University.

All study subjects were investigated for age, gender, body mass index, smoking history, the presence or absence of hypertension, diabetes mellitus, and dyslipidemia. We also checked blood levels of triglyceride, high-density lipoprotein cholesterol, and LDL cholesterol levels in all study subjects. In coronary angiography (CAG), we evaluated the location of culprit vessel, the degree of stenosis, lesion type of ACC/AHA classification, the presence or absence of thrombosis in culprit lesion, and the number of diseased vessels.

### 2.2. Study Protocol

CAG followed by PCI was performed in 40 patients with EAP, and 27 ACS patients underwent emergent PCI. Immediately after CAG or prior to PCI, we performed blood sampling from the ascending aorta and coronary sinus for measurements of soluble LOX-1 and LAB. We also measured the levels of creatine kinase (CK), CK-MB, troponin I, and hs-CRP in blood samples from the aorta. Samples were immediately prepared after collecting from patients and stored at −80°C until measurement of soluble LOX-1 and LAB. The samples from the coronary sinus were collected using a 5F multipurpose catheter (Goodman Co. Ltd., Sunnyvale, CA). When a certain number of samples were collected, we measured soluble LOX-1 and LAB levels as shown below [9].

### 2.3. Measurement of Soluble LOX-1

Serum soluble LOX-1 levels were measured as described previously [9]. Briefly, 40 *μ*L of the standard recombinant human LOX-1 or 4-fold diluted sera was applied to the 384-well plates immobilizing anti-human LOX-1 antibody (TS92, 0.25 *μ*g/well). Bound soluble LOX-1 was detected by the combination of another anti-human LOX-1 antibody (HUC5-40) and a peroxidase-conjugated donkey anti-chicken IgY (AP194P, Chemicon, Billerica, MA) with the substrate solution containing 3, 3′5, 5′-tetramethylbenzidine (TMB solution, Bio-Rad Laboratories, Hercules, CA). The soluble LOX-1 measurement range was from 62.5 to 10,000 pg/mL.

### 2.4. Measurement of LOX-1 Ligands Containing Apolipoprotein B (LAB)

Values of LAB were measured as described previously [8, 9]. Forty microliters of the standard oxidized LDL or 20-fold diluted sera was applied to the 384-well plates immobilizing recombinant human LOX-1 (61–273, 0.25 *μ*g/well). Bound LAB was detected by the combination of chicken monoclonal anti-ApoB antibody (HUC20) and a peroxidase-conjugated donkey anti-chicken IgY (AP194P, Chemicon) with TMB solution. The oxidized LDL measurement range was from 156 to 10,000 ng/mL.

### 2.5. Measurement of Other Biomarkers

Troponin I and hs-CRP concentrations were measured by commercially available electrochemiluminescent immunoassay (ECLIA, Roche Diagnostics, Berlin, Germany). Both CK and CK-MB concentrations were measured by an automated analyzer (Dimension EXL 200, Siemens, Berlin, Germany).

### 2.6. Statistical Analyses

The data between ACS and EAP groups were compared by the Mann-Whitney *U* test. The data between the aorta and coronary sinus were compared by paired *t*-test. Correlations between soluble LOX-1 and other variables were evaluated by linear regression analysis (Spearman's rank-correlation coefficient). The abilities of soluble LOX-1 and LOX-1 index to detect ACS were assessed using receiver operating characteristic (ROC) curves and expressed by the area under the curve (AUC) with 95% confidence intervals, which plots the true-positive fraction (sensitivity) against the false-positive fraction (1-specificity). A cut-off point was determined exploratively as the point on the ROC curve closest to the upper left corner. Among the parameters of the ROC curves, the significant difference was determined by Stat Flex, version 5.0 (Artech, Inc., Osaka, Japan). All data are expressed as mean ± standard deviation (SD), and skewed variables are presented as median and interquartile ranges. A level of *P* < 0.05 was considered significant.

## 3. Results

### 3.1. Patient Characteristics

Table 1 shows the comparison of the clinical characteristics between the patients with ACS and EAP. There were no significant differences in age, the percentages of hypertension, dyslipidemia, diabetes, and obesity, and the serum levels of LDL cholesterol and triglycerides between the ACS and EAP groups, whereas the ACS group had higher hs-CRP (*P* < 0.05) values and lower HDL cholesterol (*P* < 0.01) levels than the EAP group.  Table 2 shows the comparison of angiographic characteristics between the ACS and EAP groups. Culprit vessel, ACC/AHA classification, and the number of diseased vessels were not different, but the degree of stenosis was more severe (*P* < 0.001) and the number of visible thrombi was higher (*P* < 0.001) in the ACS group than in the EAP group.

### 3.2. Coronary Sinus and Aortic Levels of Soluble LOX-1

**Figure 1:**
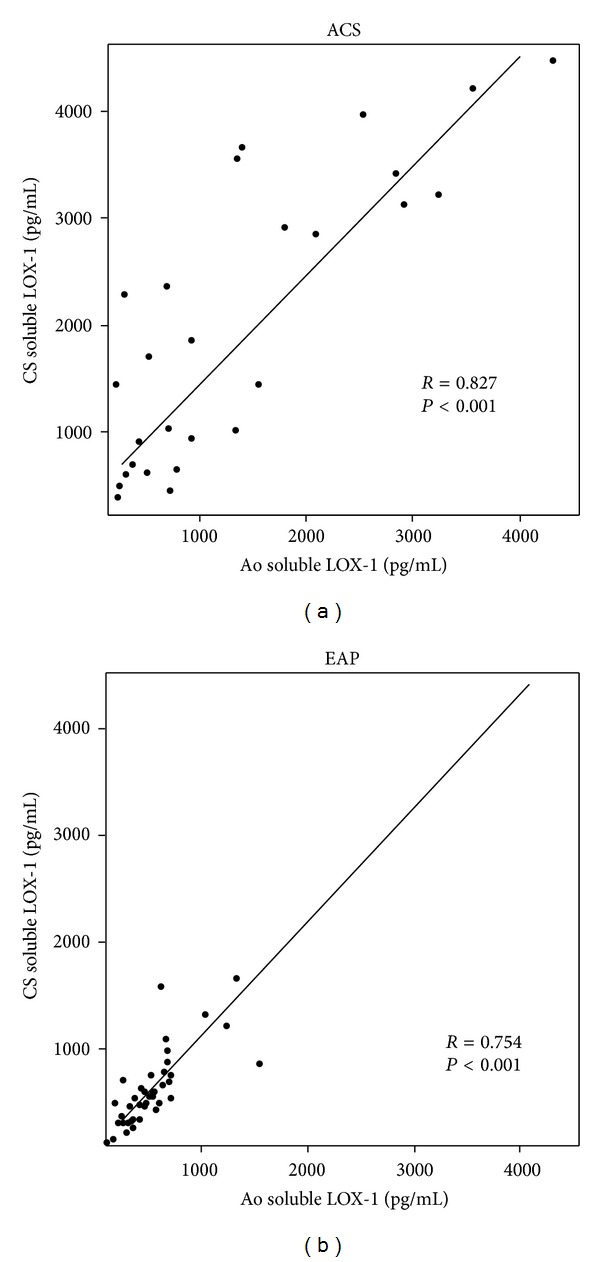
Correlations between soluble LOX-1 levels in the aorta and coronary sinus in the ACS (*n* = 27) and EAP (*n* = 40) groups as determined by ELISA. Linear regression analysis demonstrated positive correlations between soluble LOX-1 levels in the aortic and coronary sinus in the ACS (*R* = 0.827, *P* < 0.001) and EAP (*R* = 0.754, *P* < 0.001).

Regression analysis demonstrated a positive correlation in soluble LOX-1 values between coronary and systemic circulation in both ACS (*R* = 0.827, *P* < 0.001) and EAP (*R* = 0.754, *P* < 0.001) groups (Figure 1). There were no significant correlations between coronary or systemic soluble LOX-1 values and time intervals from onset in the ACS group, although the patients with short time intervals from the onset tended to show higher soluble LOX-1 levels (data not shown).

**Figure 2:**
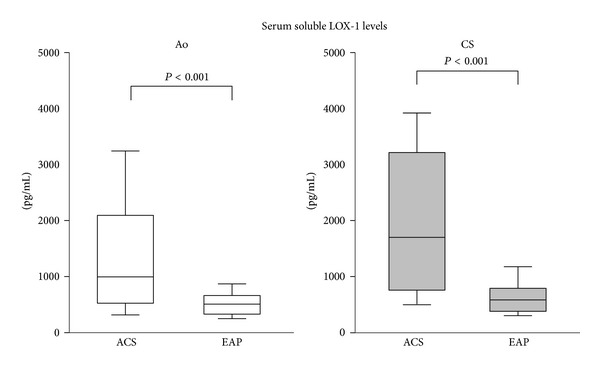
Comparisons of soluble LOX-1 levels in the aortic and coronary sinus between the ACS and EAP groups. Both the aorta and coronary sinus soluble LOX-1 levels were higher in the ACS than those in the EAP (*P* < 0.001). Center horizontal lines indicate median values; top and bottom edges of boxes, 25th and 75th percentiles; and lower and upper bars, 10th and 90th percentiles.

As shown in Figure 2, soluble LOX-1 concentrations in the coronary sinus and aorta were significantly elevated in patients with ACS compared to those in patients with EAP (aortic levels of soluble LOX-1: ACS, 980.3 (515.2–2081.9) pg/mL versus EAP, 514.1 (344.9–677.3) pg/mL; coronary sinus levels of soluble LOX-1: ACS, 1700.7 (746.7–3194.6) pg/mL versus EAP, 591.3 (401.8–803.2) pg/mL, *P* < 0.001, resp.).

**Figure 3:**
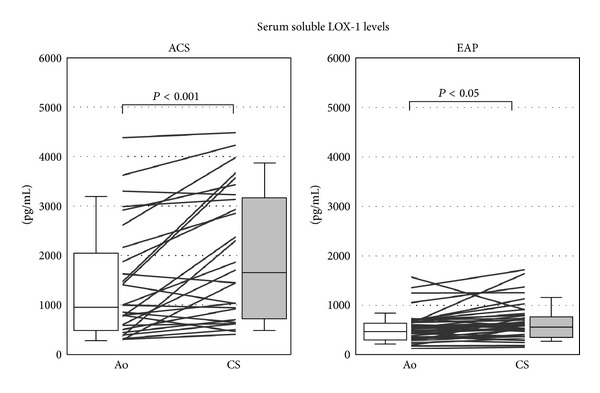
Comparison of aortic and coronary sinus soluble LOX-1 levels. Both the ACS and EAP groups had higher values of coronary soluble LOX-1 than those of the aorta (*P* < 0.001 and *P* < 0.05, resp.). Each line represents individual values of ACS and EAP patients.

Interestingly, the soluble LOX-1 levels in the coronary sinus of patients with either ACS or EAP were significantly higher than those in the aorta (ACS patients: aorta, 980.3 (515.2–2081.9) pg/mL versus coronary sinus, 1700.7 (746.7–3194.6) pg/mL; EAP patients: aorta, 514.1 (344.9–677.3) pg/mL versus coronary sinus, 591.3 (401.8–803.2) pg/mL, *P* < 0.001 and *P* < 0.05, resp., Figure 3). These data suggested that soluble LOX-1 originated from the coronary circulation and was secreted mainly from plaque in the coronary artery.

We found no correlations between soluble LOX-1 and CK-MB (*R* = 0.170, *P* = 0.53), troponin I (*R* = −0.116, *P* = 0.65), or hs-CRP (*R* = −0.119, *P* = 0.55) in the ACS group (data not shown).

### 3.3. LOX-1 Ligand and LOX-1 Index

There were no significant differences in levels of serum LAB in the coronary sinus and aorta between the ACS and EAP groups (Table 3). However, levels of LOX-1 index in the coronary sinus were significantly greater than those in the systemic circulation in both the ACS and EAP groups (*P* < 0.001, each).

### 3.4. Diagnosis for ACS

**Figure 4:**
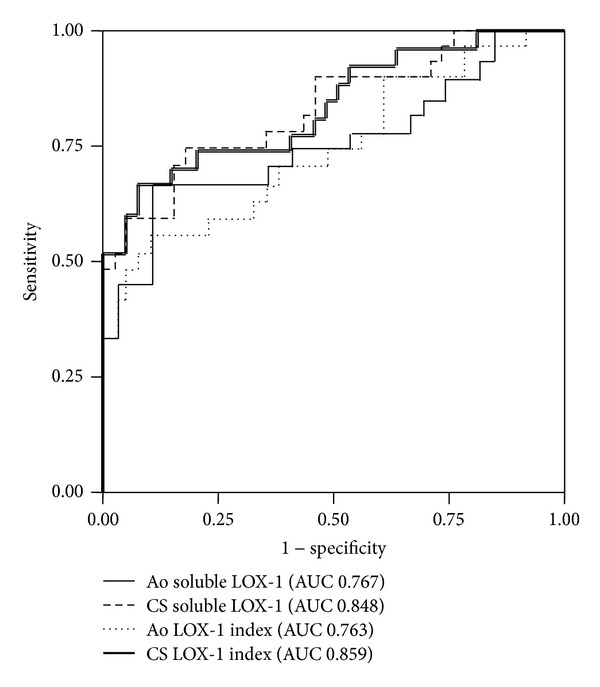
Receiver operating characteristic (ROC) curves of the aortic and coronary sinus levels of soluble LOX-1 and LOX-1 index for the ACS diagnosis. The area under the curve (AUC) values of aortic and coronary sinus soluble LOX-1 were 0.767 and 0.848, respectively, whereas AUC values were 0.763 and 0.858 for aorta and coronary sinus levels of LOX-1 index, respectively.

To compare the diagnostic sensitivity and specificity of coronary and systemic soluble LOX-1 and LOX-1 index, ROC curves were used to compare the diagnostic sensitivity and specificity of coronary and systemic soluble LOX-1 and LOX-1 index to detect ACS diagnosis.  Figure 4 shows that AUC in the coronary sinus level of soluble LOX-1 was larger than that in the aortic level of soluble LOX-1 (0.848 versus 0.767). Moreover, AUC of LOX-1 index was also higher in the coronary sinus than in the aorta (0.859 versus 0.763).

## 4. Discussion

LOX-1 is a type II membrane protein belonging to the C-type lectin family with a short N-terminal cytoplasmic domain and a long C-terminal extracellular domain [4, 12]. Various studies including ours have shown that LOX-1 also is involved in platelet-endothelium and monocyte-endothelium interactions, suggesting that LOX-1 plays an integral role in plaque formation in addition to plaque rupture [13, 14]. Importantly, LOX-1 is expressed by vascular cell components including macrophages, smooth muscle cells, and endothelial cells in human atherosclerotic lesions, and its expression is augmented by vasoactive and atherogenic stimuli [15, 16]. Moreover, a previous study demonstrated that circulating soluble LOX-1 is generated by proteolytic cleavage in the membrane proximal extracellular domain [6]. Therefore, the levels of circulating soluble LOX-1 may reflect vulnerability including protease activity of plaques in cardiovascular disease.

Hayashida et al. first highlighted the circulating levels of soluble LOX-1 as a useful biomarker in ACS [7], and they also reported no correlation between soluble LOX-1 and other biomarkers derived from damaged cardiomyocytes such as troponins and CK-MB, which was confirmed by the present study [6]. These findings suggest that elevated soluble LOX-1 derives from vulnerable plaques, which are abundant in LOX-1-expressing cells and protease activity, but not from injured cardiomyocytes in ACS, although it has been shown that cardiomyocytes may express LOX-1 [17].

In stable CAD patients, soluble LOX-1 level was higher with the increasing of diseased lesion number [18] and in proximal/mid lesion than in distal lesion of LAD [19]. Pirillo and Catapano reported that circulating soluble LOX-1 might be reflected to CAD severity [20]. A recent study using optical coherence tomography has shown that plaque rupture takes place even in coronary artery plaque in patients with stable angina pectoris [21]. In the present study, soluble LOX-1 levels increased in some cases with EAP, whose plaques might have ruptured.

We found that soluble LOX-1 levels tended to be higher in the early stage after the onset of ACS, although we did not monitor the time course profile of circulating soluble LOX-1 in the same patients. This tendency suggests that soluble LOX-1 levels rapidly increase when plaque rupture takes place, leading to a further reduction of soluble LOX-1 over the course of time. Previous investigators have clearly demonstrated that soluble LOX-1 levels rapidly decreased after PCI, suggesting that soluble LOX-1 and protease-expressing cells are destroyed by PCI [7]. These findings strongly suggest that increased enzymatic activity in vulnerable plaques augments the cleavage of LOX-1, and soluble LOX-1 is then released into the systemic circulation via the coronary circulation.

We had assumed that the LOX-1 index would be a better marker for ACS detection than soluble LOX-1 or the LOX-1 ligand, since the LOX-1 index has been shown to be associated with an increased risk of CAD in a community-based cohort study [9]. In the present study, ROC curve analysis revealed that soluble LOX-1 and LOX-1 index in the coronary sinus level might be useful in the diagnosis of ACS compared to levels in the aorta.

Study limitations include the small number of subjects in this investigation. A second limitation, as described above, is the lack of additional biomarker(s) from unstable plaques and oxidative stress, which would be crucial for the pathogenesis of ACS. These issues need to be further investigated for better understanding of the pathogenesis of CAD.

In conclusion, we found that elevated circulating LOX-1 is derived from the coronary circulation in CAD, and soluble LOX-1 and LOX-1 index were useful biomarkers for the diagnosis of ACS.

## Figures and Tables

**Table 1:** Comparisons of clinical characteristics between the ACS and EAP groups.

	ACS (*n* = 27)	EAP (*n* = 40)	*P* value
Age (years)	69 ± 12	70 ± 7	n.s.
Gender (Male/Female)	20/7	23/17	n.s.
Hypertension, *n* (%)	22 (81.4)	37 (92.5)	n.s.
Diabetes mellitus, *n* (%)	12 (44.4)	20 (50.0)	n.s.
Dyslipidemia, *n* (%)	20 (74.1)	33 (82.5)	n.s.
Current or past smoker, *n* (%)	16 (59.3)	17 (42.5)	n.s.
Obesity (BMI > 25), *n* (%)	7 (25.9)	17 (42.5)	n.s.
Blood samples data			
Triglyceride (mg/dL)	95.5 ± 54.3	112.6 ± 55.2	n.s.
HDL-cholesterol (mg/dL)	42.8 ± 8.4	49.7 ± 11.2	<0.01
LDL-cholesterol (mg/dL)	106.4 ± 36.8	101.4 ± 29.9	n.s.
Hs-CRP* (mg/dL)	0.125 (0.087–1.270)	0.052 (0.028–0.142)	<0.05

ACS: acute coronary syndrome; EAP: effort angina pectoris; BMI: body mass index; HDL: high density lipoprotein; LDL: low density lipoprotein; CRP: C-reactive protein.

*Skewed data are reported as median (inter-quartile range).

**Table 2:** Comparisons of angiographic characteristics between the ACS and EAP groups.

Groups	ACS (*n* = 27)	EAP (*n* = 40)	*P* value
Culprit vessel, *n* (%)			n.s.
Left main trunk	1 (3.7)	1 (2.5)	
Left anterior descending artery	10 (37.0)	16 (40.0)	
Left circumflex artery	8 (29.6)	10 (25.0)	
Right coronary artery	8 (29.6)	13 (32.5)	
Degree of stenosis, *n* (%)			<0.001
75%	2 (7.4)	3 (7.5)	
90%	2 (7.4)	23 (57.5)	
99%	12 (44.4)	9 (22.5)	
100%	11 (40.7)	5 (12.5)	
ACC/AHA classification, *n* (%)			n.s.
Type A	5 (18.5)	3 (7.5)	
Type B1	2 (7.4)	8 (20.0)	
Type B2	16 (59.3)	19 (47.5)	
Type C	4 (14.8)	10 (25.0)	
Visible thrombus, *n* (%)	8 (29.6)	0 (0)	<0.001
Diseased vessels, *n* (%)			n.s.
1 vessel disease	11 (40.7)	13 (32.5)	
2 vessel disease	13 (48.1)	18 (45.0)	
3 vessel disease	3 (11.1)	9 (22.5)	

**Table 3:** Comparisons of LAB and LOX index between the ACS and EAP groups.

	ACS (*n* = 27)	EAP (*n* = 40)
LAB (ng/mL)		
Aorta	349 (269–543)	331 (223–471)
Coronary sinus	480 (306–647)	347 (242–488)
LOX-1 index (10^6^)		
Aorta	0.5115 (0.1555–1.0524)	0.1713 (0.1087–0.2989)
Coronary sinus	0.9256 (0.2691–1.9087)^#^	0.2160 (0.1323–0.4005)^#^

LAB, LOX-1 ligand containing apolipoprotein B; Skewed data are reported as median (inter-quartile range), ^#^ *P* < 0.001 versus aorta in same group.
